# HIV infection and stroke: current perspectives and future directions

**DOI:** 10.1016/S1474-4422(12)70205-3

**Published:** 2012-10

**Authors:** Laura A Benjamin, Alan Bryer, Hedley CA Emsley, Saye Khoo, Tom Solomon, Myles D Connor

**Affiliations:** a Brain Infections Group, Institute of Infection and Global Health, University of Liverpool, Liverpool, UK; b Malawi-Liverpool-Wellcome Major Overseas Clinical Research Programme, Blantyre, Malawi; c Walton Centre NHS Foundation Trust, Liverpool, UK; d Division of Neurology, Groote Schuur Hospital and University of Cape Town, Cape Town, South Africa; e Royal Preston Hospital, Preston, UK; f School of Medicine, University of Liverpool, Liverpool, UK; g Tropical and AIDS Related Disease Research Group, Institute of Translational Medicine, University of Liverpool, Liverpool, UK; h NHS Fife, Kirkaldy, UK; i Division of Clinical Neuroscience, University of Edinburgh, Edinburgh, UK; j School of Public Health, University of the Witwatersrand, Johannesburg, South Africa

## Abstract

HIV infection can result in stroke via several mechanisms, including opportunistic infection, vasculopathy, cardioembolism, and coagulopathy. However, the occurrence of stroke and HIV infection might often be coincidental. HIV-associated vasculopathy describes various cerebrovascular changes, including stenosis and aneurysm formation, vasculitis, and accelerated atherosclerosis, and might be caused directly or indirectly by HIV infection, although the mechanisms are controversial. HIV and associated infections contribute to chronic inflammation. Combination antiretroviral therapies (cART) are clearly beneficial, but can be atherogenic and could increase stroke risk. cART can prolong life, increasing the size of the ageing population at risk of stroke. Stroke management and prevention should include identification and treatment of the specific cause of stroke and stroke risk factors, and judicious adjustment of the cART regimen. Epidemiological, clinical, biological, and autopsy studies of risk, the pathogenesis of HIV-associated vasculopathy (particularly of arterial endothelial damage), the long-term effects of cART, and ideal stroke treatment in patients with HIV are needed, as are antiretrovirals that are without vascular risk.

## Introduction

The incidence of stroke has increased by 100% in middle-to-low-income countries in the past 10 years.^1^, ^2^ Although much of this increase is probably related to the increasing burden of vascular risk factors and the ageing population, infectious causes of stroke might also contribute.^3^, ^4^ Low-income and middle-income countries have the highest incidence of HIV infection,^5^ therefore occurrence of the two disorders in one patient might often be coincidental. However, HIV infection potentially affects stroke risk and cause, and HIV treatment can result in vascular damage, perhaps conferring an additional risk.^6^, ^7^

Between 1% and 5% of patients with HIV develop stroke in clinical series, although a higher proportion (4–34%) have cerebral ischaemic lesions at autopsy.^8^, ^9^, ^10^, ^11^, ^12^, ^13^, ^14^ There was little correlation between pathological evidence of cerebral ischaemic lesions and clinical manifestations before death in series that assessed this.^13^, ^15^ In the USA, admissions of patients with stroke and concurrent HIV infection have increased by 43% over 9 years.^16^ Despite this apparent association, surprisingly little research has assessed the effect of HIV infection on the burden and nature of stroke, such as the extent to which HIV increases stroke risk, and the pathogenesis of stroke in individuals with HIV.^6^, ^17^

Standard HIV treatment—combination antiretroviral therapy (cART)—might also contribute to the risk of stroke, directly by accelerating atherosclerosis and indirectly by increasing life expectancy.^18^ Inevitably, exposure to conventional vascular risk factors (eg, ageing, hypertension, diabetes, hypercholesterolaemia, and cigarette smoking) will continue to increase as the HIV population lives longer.^19^ Furthermore, the continuous exposure to HIV, albeit at lower viral titre, and low-grade chronic systemic inflammation might add to the risk of stroke.^20^, ^21^

Assessment of the effect of HIV infection on stroke has public health relevance, particularly with respect to the increased frequency of stroke in regions of high HIV prevalence. From a practical clinical perspective, when physicians in all regions are managing a patient with HIV who has had a stroke, they need to know the extent to which the HIV infection and its treatment might affect the cause, clinical presentation, and management of the stroke, but they also need to consider that stroke might be the presenting feature of HIV infection in patients whose HIV status is not known.

In this Review we describe what is known about the risk of stroke in people infected with HIV. We present emerging theories of the mechanism of stroke in these patients and discuss the effect of HIV on clinical stroke syndromes and implications for the management of stroke in HIV-infected patients.

## HIV infection and the risk of stroke

Both HIV infection and cART could potentially increase an individual's risk of stroke; therefore, any assessment of the risk attributable to HIV should ideally have been done in an untreated population. Most studies have been retrospective or have compared unmatched groups or cohorts or assessed the prevalence of HIV infection in stroke series compared with the general population of a similar age. Most have been hospital-based series, and some studies have compared cerebral infarction (rather than clinical stroke) in HIV-infected and HIV-unaffected brains at autopsy. No case-controlled or cohort studies have prospectively assessed the risk of stroke in HIV-infected populations, and so the evidence for an increased risk with HIV infection is based on very little data.

### Pre-cART era

A retrospective hospital-based case-control study compared 113 patients aged 19–44 years who had had a stroke with 113 age-matched and sex-matched patients with asthma and known HIV status in the USA between 1990 and 1994, and reported that HIV infection was associated with a doubling of the risk of stroke (odds ratio [OR] 2·3, 95% CI 1·0–5·3). The risk was higher for cerebral infarction (OR 3·4, 95% CI 1·1–8·9) than for cerebral haemorrhage (OR 1·3, 95% CI 0·3–6·4). 11 (44%) of 25 patients with HIV who had had a stroke had a coagulopathy (protein S deficiency) or meningitis.^22^ A population-based study from Baltimore, MD, USA (1988–91) that identified 12 patients who had had a stroke and had AIDS (ascertained retrospectively) reported that AIDS conferred an adjusted relative risk of 13·7 (95% CI 6·1–30·8) for ischaemic stroke and 25·5 (95% CI 11·2–58·0) for cerebral haemorrhage.^23^ The selection of (by definition) patients with advanced HIV infection who would therefore have a particularly high risk of stroke, and under-reporting of AIDS in the study population, might have resulted in the surprisingly high relative-risk ratios in this study.^17^

Not all studies have reported an association between HIV infection and stroke. A 1980's autopsy study from the USA retrospectively compared the brains of patients aged 20–50 years with and without AIDS. Cerebrovascular disease was present in 13 (8%) of 154 patients with AIDS, and in a higher proportion (25 [23%] of 111) of controls, which suggested that stroke was not more common in patients with AIDS at autopsy.^12^ A hospital-based study in KwaZulu-Natal, South Africa, reported that the prevalence of HIV infection in a series of patients younger than 50 years who had had a stroke was 16%, similar to the prevalence of HIV in the general population of a similar age.^24^ However, the extent to which this result can be generalised to the population of KwaZulu-Natal is unclear, because only 12% of patients with stroke included in the Durban Stroke Data Bank were black Africans, compared with a community proportion of 85%.^25^ In a retrospective hospital-based study of 293 black African patients with stroke aged 15–44 years from the same region as the previous study, HIV infection (present in 56) increased the risk of ischaemic stroke (OR 2·3, CI 0·8–7·7; p=0·09), although this finding was not statistically significant.^26^ Patients with opportunistic infection were excluded from the study. However, HIV serology was not assessed in almost a third of patients.

### Post-cART era

No studies have prospectively assessed the effect of HIV infection on stroke risk since the introduction of cART.^6^, ^7^ A retrospective analysis of hospital admission data for all individuals with HIV in Denmark (1995–2010) established the rate of cerebrovascular events and compared it with the rate in a sex-matched and age-matched general population, stratified by intravenous drug use (IDU) and conventional vascular risk factors. Individuals with HIV had an increased risk of cerebrovascular events compared with controls (non-IDU HIV-adjusted incidence rate ratio 1·60, 95% CI 1·32–1·94). Risk of cerebrovascular events increased when associated with IDU, low CD4 count before cART, and abacavir.^27^ Although, arguably, this study provides the best evidence linking HIV with stroke risk, the retrospective nature of the study and absence of detailed prospective clinical assessment might have led to misclassification of cerebrovascular events in a population in which stroke mimics are common.

In a large US study that assessed population-wide hospital stroke discharge diagnoses over 9 years (1997–2006), the number of patients with HIV admitted for stroke rose by 43%, after adjustment for population size. This increase was associated with a total increase in the proportion of patients who had an ischaemic stroke in the HIV population, and also coincided with the introduction of cART in the mid-1990s.^16^ How much of this rise was due to an increase in the incidence of HIV infection, to the effect of cART on stroke risk in people with HIV, to an increased incidence of stroke in HIV-positive individuals, to improved survival of those with HIV, or indeed to better recognition of stroke symptoms is impossible to tease out. However, the increase in HIV-associated stroke admissions occurred at the same time as an overall (7%) reduction in all stroke admissions, suggesting, but not substantiating, an association between HIV infection and stroke.

## Clinical presentation of stroke in patients with HIV

### Age and stroke

Individuals with stroke who are HIV positive are reported to be younger than patients with stroke who do not have HIV, which could be a result of the age of the population at risk of HIV infection or a sign that the mechanism of stroke in HIV is largely independent of classical vascular risk factors. Ovbiagele and Nath^16^ reported a median age for individuals with HIV who had had a stroke of 42·9 years in 1997 and 48·4 years in 2006 in the previously described retrospective analysis of hospital discharges in the USA, and Ortiz and colleagues^28^ reported a median age of 42 years in a similar setting between 1997 and 2002. However, in lower-income countries such as South Africa and Malawi, patients with HIV who have strokes are younger—eg, in Cape Town, South Africa (2000–06) the median age was 33·4 years,^29^ and in Blantyre, Malawi (2008–09) it was 39·8 years.^30^ One possible explanation for this regional variation and the increase in median age seen in the USA over a decade might be the use of cART, perhaps because cART delays the time to stroke onset.

### Clinical descriptions of stroke

Clinical descriptions of stroke in patients with and without HIV are similar.^31^, ^32^, ^33^ Although the sudden onset of a focal neurological deficit is typical, atypical stroke presentations are common in the context of HIV infection—eg, acute confusion, fever, acute loss of consciousness, and stepwise focal neurological presentation over hours to days.^34^, ^35^, ^36^ Alternative causes or mimics of stroke should be excluded before a final diagnosis of HIV-associated stroke is made.

### Stroke type and subtype

Ischaemic stroke seems to be more frequent than cerebral haemorrhage in patients with HIV, at least in hospital-based series from sub-Saharan Africa, where it is reported in over 90% of HIV-associated strokes.^28^, ^33^, ^37^ This trend was not supported by the scientific literature from the pre-cART era in the USA, with near equal proportions of cerebral haemorrhage and ischaemic infarction in the Baltimore study described previously, although this result was probably due in part to illicit drug use or hospital-admission bias.^23^ However, Ovbiagele and Nath^16^ reported that ischaemic stroke was the predominant pathological stroke type and that the proportion of patients with ischaemic stroke among those with HIV doubled between 1997 and 2006.^16^ Whether this rise is a result of cART or methodological factors is not clear.

In a South African hospital-based study, the subtypes of ischaemic stroke, classified according to Oxfordshire Community Stroke Project (OCSP) criteria, in the HIV-positive group (n=64) were lacunar stroke (n=13), partial anterior circulation stroke (n=33), total anterior circulation stroke (n=11), and posterior circulation stroke (n=7).^29^ The proportions of these subtypes follow similar trends to the original OCSP (lacunar stroke 25%, partial anterior circulation stroke 34%, total anterior circulation stroke 17%, and posterior circulation stroke 24%), but posterior circulation stroke was less frequent and partial anterior circulation stroke more frequent in this HIV population than in the OCSP, although this finding was based on a small number of patients.^38^ However, the proportions of stroke type and subtype will only be determined accurately in a community-based HIV and stroke population because hospital admission favours cerebral haemorrhage.

## Causes of stroke in patients with HIV

HIV infection can potentially cause stroke in many ways: indirectly through cardioembolism, coagulopathy, or non-HIV infective vasculitis; or directly through HIV-associated vasculopathy. Panel 1 lists the mechanisms that might result in an ischaemic or haemorrhagic stroke.

### Coagulopathy

HIV infection might predispose an individual to both arterial and venous thrombosis; however, to what extent this predisposition is caused by coagulopathy is unclear. Deficiencies in protein C and protein S are sometimes associated with intracranial venous thrombosis, but rarely with arterial stroke in adults without HIV.^39^, ^40^ Although deficiencies in protein C and protein S have been identified in HIV-infected patients who have had a stroke, whether these deficiencies are secondary events or caused directly by the HIV infection is unclear. In one South African case series in which patients with stroke and HIV infection (n=33) were compared with an unmatched group of patients with stroke who were not HIV-positive (n=33), the proportion of patients with protein S deficiency did not differ significantly between the groups.^37^

Antiphospholipid antibodies have been described infrequently in people with HIV who have had a stroke, but as transient raised antiphospholipid antibody titres are often reported with viral infection, raised concentrations should only be regarded as clinically relevant in patients meeting the International Haematology Consensus statement criteria for antiphospholipid syndrome.^41^ These criteria have only rarely been applied in studies investigating stroke and HIV infection, and the true frequency of any association is unclear.^33^, ^42^, ^43^ Factor V Leiden is not associated with HIV-related stroke.

### Cardioembolism

Cardioembolism accounts for 4–15% of ischaemic strokes in people with HIV.^24^, ^28^, ^42^ HIV-associated dilated cardiomyopathy is a frequently reported cause of cardiac disease, particularly in sub-Saharan Africa.^44^, ^45^, ^46^ This disorder might be associated with opportunistic infection and has been attributed to HIV itself, although its pathogenesis is uncertain. We have therefore used a deliberately vague term, HIV-associated cardiac dysfunction, in panel 1. Assessment of cardioembolic causes of stroke in patients with HIV in studies from sub-Saharan Africa is further complicated by the high prevalence of non-ischaemic cardiomyopathy and rheumatic heart disease in people without HIV.^47^ Other potential causes of cardioembolism include bacterial and marantic endocarditis, and ischaemic heart disease.^48^, ^49^

### Opportunistic infections

Some infections are well established causes of stroke. *Mycobacterium tuberculosis*, syphilis, and varicella zoster virus cause stroke in patients without HIV, but immunosuppression caused by HIV increases susceptibility to acquisition or reactivation of these infections.^50^, ^51^, ^52^ Tuberculosis-related stroke is thought to be a complication of tuberculous meningitis, and focal signs masquerading as stroke might be the first manifestation.^50^, ^53^ Therefore, actively looking for tuberculosis in patients with HIV who have had a stroke (who are more at risk than those without HIV infection who have had a stroke) is important. Varicella zoster virus infection might cause cerebral vasculitis and stroke in immunosuppressed patients, although the skin manifestation can be absent at the time of presentation in about a third of patients with stroke, making diagnosis difficult.^51^, ^54^, ^55^ Co-infection with HIV compounds the diagnosis of neurosyphilis, which is another potential cause of stroke.^52^, ^56^ Furthermore, an increase in meningovascular complications has been described in people with HIV.^52^, ^56^, ^57^

Cytomegalovirus and *Candida albicans* infections have been associated with HIV infection and stroke in a few case series, but further evidence is needed to establish whether they are a cause of stroke in HIV-infected patients.^8^, ^58^ cART might unmask occult opportunistic infections that subsequently cause a stroke. This possibility should be considered in all patients who have had an acute stroke or have worsening of stroke symptoms after initiation of cART.^58^, ^59^, ^60^, ^61^ Even in high-income countries, opportunistic infection still leads to substantial mortality, despite the use of cART and good diagnostic facilities.^60^ Furthermore, infections outside the CNS might contribute to the development of a prothrombotic state, potentially increasing the risk of ischaemic stroke.^4^

### HIV-associated vasculopathy

**Figure 1:**
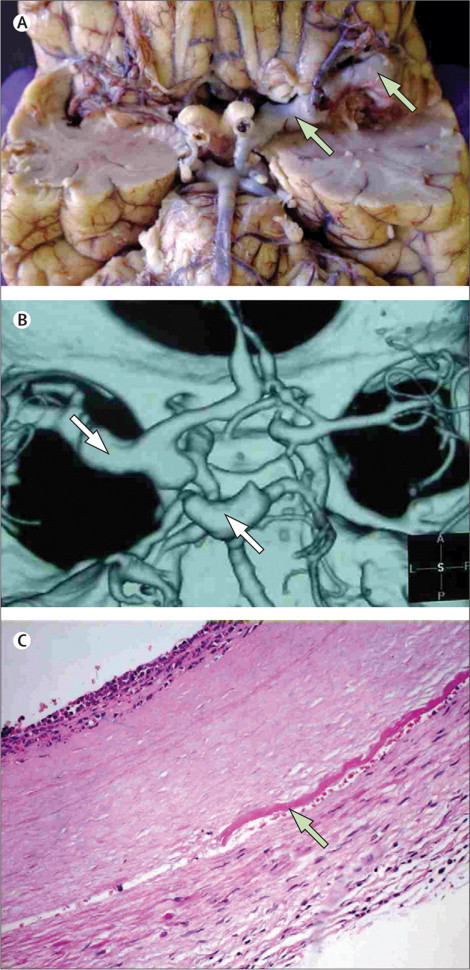
Aneurysmal HIV-associated vasculopathy (A) View of the ventral surface of the brain showing a thrombotic occlusion of the left internal carotid artery and evidence of vasculopathy of the left middle cerebral artery (arrows). (B) CT angiogram showing several fusiform aneurysms affecting the circle of Willis. (C) Haematoxylin and eosin stain of a cross-section through an aneurysmal cerebral artery showing evidence of intimal hyperplasia, fragmentation of internal elastic lamina (arrow), and neutrophil infiltrate; note the absence of atherosclerosis. Reproduced from Tipping and colleagues,^33^ by permission of the American Medical Association.

HIV-associated vasculopathy is a relatively new and evolving term used to describe several arterial changes associated with HIV infection (figure 1). Some investigators have distinguished aneurysmal arterial changes from vasculitis and others have suggested that in view of the uncertainty regarding the pathogenesis of vasculitis, all arterial abnormalities thought to be associated with stroke should be regarded as part of an HIV-associated vasculopathy. A working definition would, in our opinion, be useful to both clinicians and pathologists. On the basis of the current scientific literature, we propose that the term HIV-associated vasculopathy should include any abnormality of the intracranial or extracranial cerebral blood vessels that results directly or indirectly from HIV infection, but should exclude vasculitis associated with opportunistic infection or neoplastic involvement of the vessels (panel 1). Aneurysmal dilatation in HIV-infected patients can be extracranial, involving the carotid, aorta, iliac, and other large arteries, or intracranial, involving branches of the circle of Willis.^62^, ^63^, ^64^ In a series of patients with HIV and stroke (n=64), seven patients had an extracranial non-aneurysmal vasculopathy that manifested as either stenosis or occlusion of the internal carotid artery. Autopsy of one of the individuals who died from complications associated with their stroke showed microscopic evidence of neovascularisation and vessel wall inflammation with a thrombus occluding the lumen. Six patients had radiological evidence of intracranial vasculopathy, typically involving the medium-sized vessels with or without fusiform aneurysm or stenosis. One patient's histology showed luminal thrombosis, concentric intimal hyperplasia with hyalinisation, and fragmentation of the elastic lamina.^29^ In this case series, patients with an extracranial vasculopathy had a significantly higher CD4 count compared with those with intracranial vasculopathy.^29^ Patients with HIV infection and stroke in this study were young (mean age 33·3 years) and did not have evidence of atherosclerosis.

Panel 1 **Possible HIV-related causes of stroke**

**Ischaemic**

*HIV-associated vasculopathy (abnormality of the cerebral blood vessels as a direct or indirect result of HIV infection, but excluding opportunistic infection vasculitis)*

• Associated with aneurysm formation (either intracranial or extracranial)
• Vasculitis (as a direct result of HIV infection, excluding opportunistic infection)
• Accelerated atherosclerosis
• Other disease of cerebral blood vessels associated with HIV infection (including small vessel disease changes and altered vasoreactivity)

*Opportunistic infection or neoplasia*

• Opportunistic infection causing stroke (eg, tuberculous meningitis, varicella zoster virus vasculitis, meningovascular syphilis)
• Neoplasia, such as lymphoma involving cerebral blood vessels

*Cardioembolism*

• Bacterial endocarditis
• Marantic endocarditis
• HIV-associated cardiac dysfunction
• Ischaemic heart disease

*Other established cause*

• Coagulopathy (eg, antiphospholipid syndrome)
• HIV-associated hyperviscosity

**Haemorrhagic**

• HIV-associated vasculopathy (aneurysm or vasculitis-associated)
• HIV-associated thrombocytopenia
• Mycotic aneurysm (secondary to bacterial endocarditis)

In other studies, patients with HIV and extracranial carotid aneurysms (but without stroke) had evidence of leucocytoclastic vasculitis of the vasa vasorum and periadventitial vessels without evidence of atherosclerosis or infection on culture of the blood or aneurysm wall.^63^, ^65^ Whether this form of vasculitis is an initiating or pivotal event in the development of extracranial carotid artery aneurysm formation in HIV infection or is one of several factors is not clear. Fusiform intracranial cerebral aneurysms have been described in children, usually as incidental findings, but in some they are associated with subarachnoid haemorrhage or hemiparesis.^66^, ^67^

The term vasculitis should ideally be reserved for the histologically confirmed appearance of inflammatory cells in the blood vessel wall together with associated wall damage. In early pathological studies that did not exclude all potential causes of infective vasculitis other than HIV, cerebral vasculitis attributed to HIV was probably overestimated.^13^, ^68^ However, a heterogeneous array of extracerebral vasculitis of small, medium, and large arteries has been described in patients with HIV.^65^ Although HIV antigen and particles were identified in perivascular cells in two patients, and HIV-like particles that were suggestive of a direct HIV infection (at least of perivascular tissue) have been reported in another, a direct role of HIV in the development of vasculitis is far from certain.^65^, ^69^ Other postulated mechanisms for vasculitis include immune deposition and indirect damage caused by T-cell-derived growth factors and cytokines.^65^ In clinical practice, histology is seldom available and the diagnosis of cerebral vasculitis is usually based on clinical and radiological findings and exclusion of other possible causes. In several clinical case series, HIV-associated arterial vasculitis (attributable to opportunistic infection or other known cause) accounted for 13–28% of ischaemic stroke cases.^28^, ^29^, ^42^ In a patient presenting with clinical and radiological features typical of vasculitis, in whom all other probable causes have been excluded, HIV-associated vasculitis is the most reasonable diagnosis.

HIV infection can be associated with acceleration of large-vessel atherosclerosis, potentially caused by cART and associated metabolic complications such as dyslipidaemia, insulin resistance, or diabetes, by low-grade chronic systemic inflammation, or by co-infections such as hepatitis C or cytomegalovirus.^7^, ^70^ Interactions between co-infections and cART in the context of HIV might be more complex than appreciated. For example, mitochondrial genomics might play an important part in the development of metabolic disorders and cardiovascular diseases in patients taking cART who are co-infected with HIV and hepatitis C virus.^71^ Little is known about the role of specific co-infections in atherogenesis in individuals with HIV. The possible role of an infectious burden of several microorganisms (eg, *Chlamydia pneumoniae*, *Helicobacter pylori*, cytomegalovirus, herpes viruses 1 and 2), rather than individual co-infections, should be considered, because this seems to be a relevant factor in the development of atherosclerosis in individuals without HIV.^72^

Other abnormalities of cerebral blood vessels such as altered vasoreactivity and small-vessel abnormalities have been described,^7^, ^13^ but their importance in clinical stroke is not clear.

### Other causes

Although arterial changes in patients with HIV might be associated with HIV or opportunistic infection (eg, varicella zoster virus or tuberculosis), other unrelated causes such as connective tissue disease and an arterial dissection should also be considered in this young population. Mycotic aneurysm resulting from bacterial endocarditis, HIV-induced thrombocytopenia, and complications of aneurysmal and vasculitic HIV-associated vasculopathy might result in cerebral or subarachnoid haemorrhage (panel 1).

## Pathogenesis of vasculopathy and atherogenesis in HIV

Much of our knowledge about the potential pathogenesis of HIV infection and stroke comes from experimental studies of in-vitro human brain microvascular endothelial cells, human umbilical vascular endothelial cell models, and HIV-transgenic animal models.^73^, ^74^, ^75^ In-vivo studies in man have used various imaging modalities (eg, carotid and transcranial doppler, MRI, and angiography) to show endothelial integrity indirectly. Additionally, the use of circulating biomarkers has provided some insight into endothelial dysfunction in HIV infection.^76^

**Figure 2:**
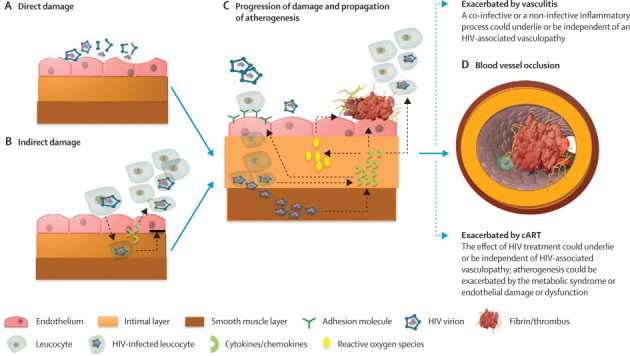
Hypotheses on the mechanism of HIV-associated vasculopathy Mechanisms are specific to atheroma and potentially applicable to the other forms of HIV-associated vasculopathy. (A) Direct damage can occur through continuous exposure of the endothelium to HIV virion or viral particles (eg, GP120 or TAT) leading to endothelial dysfunction. (B) Indirect damage can arise from circulating infected monocytes freely transmigrating the endothelium as part of a normal surveillance, with an impaired reverse transmigration, thus increasing the subendothelial population of HIV infected monocytes. The release of chemokines such as CCL2 from infected leucocytes attracts more leucocytes. (C) Several events lead to the progression of damage and propagation of atherogenesis: upregulation of cell adhesion molecules (eg, selectins), leading to increased adhesion of infected or non-infected leucocytes; release of HIV virions into the arterial smooth muscle and continued active replication of the virus in smooth muscle cells; inflammatory cytokine release from HIV-infected cells, leading to further recruitment and adhesion of leucocytes, increased production of reactive oxygen species, and derangement of the coagulation system, favouring a prothrombotic state. Underlying this continuing process is the remodelling of the vessel wall, involving intimal hyperplasia and fragmentation of the elastic lamina. (D) Thrombotic occlusion of the vessel wall lumen is one of the outcomes of this process.

The vascular endothelium is a protective barrier, preventing arterial wall inflammation, coagulation, remodelling, and ultimately, in some instances, stroke. Dysfunction of the endothelium is pivotal to the initiation and progression of vascular disease and might lead to occlusive thrombotic events mediated by leucocyte recruitment, platelet adhesion and aggregation, blood-clotting activation, and fibrinolysis derangement.^73^, ^77^ This endothelium dysfunction underpins the inflammatory process of atherosclerosis, and similar effects of endothelial dysfunction have been reported in HIV experimental models and in individuals with stroke (figure 2).^77^, ^78^

### Role of inflammation

Although HIV-1 is unlikely to be vasculotropic, the virus affects endothelial homoeostasis and function in important ways that could initiate and propagate atherogenesis. The vascular endothelium is continually exposed to stimuli such as HIV-1-infected cells (CD4+ T cells, monocytes, and macrophages), freely circulating HIV-1 viruses, HIV-1 proteins (ie, TAT and GP120) that are released with host cell lysis or actively secreted, and viral-induced proinflammatory cytokines.^73^, ^79^ All these factors potentially activate the endothelium, damaging and increasing its permeability, and thereby assisting leucocyte invasion into the vessel wall and chronic inflammation.

HIV-1-induced cytokine endothelial activation might result in the production of reactive oxygen species, the expression of cell adhesion molecules (CAMs), and the release of chemoattractants at localised areas of vascular inflammation. Akin to the pathogenesis of atherosclerosis, chemoattractants such as chemokine ligand 2 (CCL2) might recruit leucocytes to the brain during HIV infection.^80^, ^81^ CCL2 is also associated with an increase in HIV viral burden.^82^ Plasma concentrations of VCAM1, ICAM1, and E-selectin, in addition to other markers of inflammation (high-sensitivity C-reactive protein [hsCRP], interleukin 6, and the cystatins) are significantly increased in HIV-1-positive patients compared with controls.^73^, ^83^, ^84^, ^85^ The activation of these biomarkers implicates HIV-1 virus in dysfunction of the endothelium and inflammation. Endothelial cell molecules involved in coagulation, such as von Willebrand factor, thrombomodulin, plasminogen activator inhibitor-1 antigen, tissue factor, and d-dimer are deranged in HIV infection, favouring a prothrombotic state.^84^, ^85^ Furthermore, some of these biomarkers of endothelial dysfunction and inflammation positively correlate with anti-p24 antibody concentrations and disease severity.^84^, ^86^ This propagated inflammatory process could potentially accelerate atherosclerosis or independently initiate vascular disease.

Monocytes continually migrate from the bloodstream across the vascular endothelium for systemic immune surveillance and maintenance of macrophage populations in the brain. CD163 is a novel marker of activated monocytes. CD163 concentration is increased and associated with non-calcified coronary plaque in patients with HIV, suggesting a role in the pathogenesis of HIV-associated atherogenesis.^87^ Monocytes can perform reverse transendothelial migration across the endothelium, necessary for the movement of tissue monocytes or macrophages back into the bloodstream. HIV-1-infected macrophages have a reduced capacity to emigrate from the subendothelial extracellular matrix in vitro, suggesting that the infected monocyte can easily move from the blood to brain tissue, but not the reverse.^88^ Clinically, such an adaptation could promote the establishment of viral reservoirs and increase the exposure of HIV-1 and its viral proteins to the vessel wall. Since the vascular bed serves as the interface between the entry and exit of these monocytes, this could conceivably be where the greatest damage occurs. In the context of vascular disease, once the virus is subendothelial it can then infect coronary arterial smooth muscle in a manner dependent on CD4, chemokine, and endocytosis, and actively replicate in its new niche.^80^, ^89^

### Vessel wall remodelling

Animal models have shown a plausible link between endothelial dysfunction and vasculopathy. The well characterised so-called murine AIDS model of retroviral infection time-dependently decreased maximum aortic contractile responses and impaired endothelium-dependent relaxation.^90^ Isolated aortas from infected mice also expressed higher concentrations of ICAM-1 and VCAM-1.^75^ These models also showed independent HIV-associated vasculopathy.^74^, ^91^ Thus, the murine AIDS model strongly suggests that retroviral infection, similar to that of HIV-1 in man, is capable of causing vasculopathy and endothelial dysregulation in mice.

Some of these experimental findings have been corroborated in man. Brachial endothelium-dependent flow-mediated dilatation and carotid intima-media thickness (cIMT) are functional and structural indicators of endothelial integrity, and when impaired and increased, respectively, are associated with immunosuppression or increasing HIV viral concentrations.^92^, ^93^, ^94^ Suppressed HIV replication is associated with less cIMT progression (a marker of subclinical atherosclesosis).^95^ Furthermore, thinning of the arterial media layer, a possible preclinical stage of HIV vasculopathy, has been reported.^96^

HIV-1-associated endothelial dysfunction probably compromises the CNS, although whether this results in hard clinical outcomes such as stroke is unclear. HIV could plausibly initiate injury to the vascular tree or contribute to further injury to an already damaged vascular tree caused by atherosclerosis, predisposing an individual to stroke.

## Role of cART in the pathogenesis of cerebrovascular disease and stroke

Stroke risk is not only associated with HIV infection, but also with its treatment. cART could cause both direct tissue injury to arteries—resulting in raised concentration of markers of endothelial dysfunction—or indirectly cause injury through lipid modification;^97^ however, how relevant this is to cerebrovascular disease is still uncertain. An increase in the concentration of biomarkers indicating endothelial dysfunction should be interpreted with caution, as this increase could result from other factors—namely, autoreactive cell destruction by an improved autoimmune system or the ability of the therapy to destroy cells.

In the short term, cART might reduce the risk of ischaemic stroke and transient ischaemic attack, although this theory is based on retrospective data from only one study.^98^ However, evidence to suggest that long-term cART results in endothelial toxicity and vascular dysfunction is increasing.^83^, ^98^ Non-nucleotide reverse transcriptase inhibitors and protease inhibitors, but not nucleotide reverse transcriptase inhibitors, are thought to cause inflammation and might increase cardiovascular risk. Again, little is known about the effect on cerebrovascular risk. Abacavir, a nucleotide reverse transcriptase inhibitor, has been associated with an increased concentration of hsCRP and interleukin 6, and in a retrospective cohort study, an increase in cerebrovascular events in people with HIV.^27^, ^79^ However, a meta-analysis of published and unpublished randomised controlled trials did not support an increased risk of cardiovascular events with abacavir-containing cART regimens compared with other cART. The extent to which these findings can be extrapolated to cerebrovascular disease is unknown.^99^ More research is needed, specifically with cerebrovascular events as an endpoint.

cART does not stop HIV-related endothelial dysfunction and inflammation. Ross and colleagues^83^ showed enhanced endothelial activation, inflammation, and increased cIMT in patients with HIV taking cART, concluding that although cART does reduce the virulence of HIV, it has little anti-inflammatory potential on the endothelium. Therefore, individuals with HIV receiving cART are likely to live longer, but with long-term endothelial and metabolic challenges, increasing stroke risk.

## Management

Stroke mimics are not uncommon in people with HIV. Of the 98 consecutive patients presenting with an acute focal neurological deficit in Malawi, 11 were reported to have another cause for their presentation (eg, toxoplasmosis, neurocysticercosis, tuberculoma, brain tumour).^31^ Brain imaging in patients with HIV presenting with sudden-onset focal signs and features suggestive of stroke is therefore essential for diagnosis and to distinguish between cerebral haemorrhage and ischaemic stroke.

Once a diagnosis of stroke is verified, management should be directed towards acute stroke treatment, establishment of the cause of the stroke, management of HIV infection, and secondary prevention of stroke. Concomitant HIV infection raises doubt about extrapolation from evidence-based treatment of non-HIV-related stroke. The role of intravenous thrombolysis is uncertain in the absence of randomised controlled studies in HIV-related stroke. Often, the patient's HIV status is not known at the time of presentation of acute stroke, and the decision of whether to give thrombolysis must be made within a short timeframe. Although no clear evidence of harm exists, and individuals with HIV might well have a stroke that is unrelated to HIV infection, the pathogenesis of stroke can include HIV-associated vasculopathy, infective vasculitis, infective meningitis, and other causes that might increase bleeding risk (panel 1). Reassuringly, isolated reports of successful use of thrombolysis to treat myocardial infarction in individuals with HIV are available.^100^ However, the extent to which these findings can be generalised to patients with potentially diseased cerebral vessels and higher bleeding risk with thrombolysis is unclear. Until such data become available, acute therapy, including the use of thrombolysis, will have to be decided on an individual basis, taking into account clinical judgment and patient choice.

**Figure 3:**
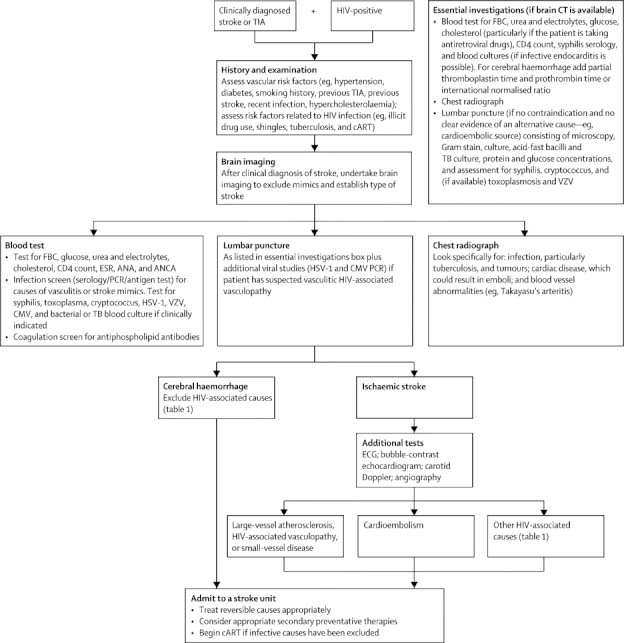
Management approach for HIV-infected patients with stroke TIA=transient ischaemic attack. cART=combined antiretroviral therapy. FBC=full blood count. ESR=erythrocyte sedimentation rate. ANA=antinuclear antibodies. ANCA=antineutrophil cytoplasmic antibodies. HSV-1=herpes simplex virus type 1. VZV=varicella zoster virus. CMV=cytomegalovirus. TB=tuberculosis. ECG=electrocardiograph.

Investigation should be directed at assessment for conventional causes of stroke and causes associated with HIV (described above and in figure 3), with particular emphasis on the identification of treatable causes. The approach to management outlined in figure 3 will not be possible in many areas where HIV infection is prevalent. In low-resource settings, investigation and treatment should be directed at identification of treatable causes of stroke or stroke mimics, such as tuberculosis, cryptococcus, toxoplasmosis, varicella zoster, or herpes virus infection, perhaps by combining CT brain scan, chest radiograph, lumbar puncture (if not contraindicated and in the absence of an alternative cause—eg, an obvious cardioembolic source), and selected blood tests (figure 3). Ancillary tests might be needed to establish the cause of stroke (eg, sputum and CSF samples for tuberculosis).^101^, ^102^ Measuring intrathecal IgG against varicella zoster virus together with CSF DNA PCR improves the likelihood of identifying this potential cause.^54^ Diagnosis of neurosyphilis in patients with HIV can be complex. A positive CSF venereal disease research laboratory test can help—when this test is negative in a patient with HIV, a test for CSF treponemal antibodies seems a reasonable approach.^52^, ^103^

Antineutrophil cytoplasmic antibodies (ANCA) assessed by immunofluorescence and enzyme-linked immunosorbent assay have been identified in patients with HIV, but not necessarily in patients with vasculitis, autoimmune disease, or specific opportunistic infection.^104^, ^105^, ^106^ Diagnosis of cerebral vasculitis in patients with HIV should be based on the results of appropriate radiological and, if possible, histological features, without the presence of any other potential cause of vasculitis (eg, opportunistic infection). In this setting, detection of ANCA might strengthen the diagnosis.

In high-resource settings, investigation should include detailed assessment of cerebral arteries with carotid doppler and CT, or magnetic resonance angiography, depending on local skills. In selected patients, perhaps those who have had further events or have a comorbidity suggesting autoimmune or other disease, conventional angiography or brain biopsy might be indicated. In low-resource settings without access to brain imaging, we advise basic stroke care with the approach outlined in the South African stroke guideline.^107^

The role of immunosuppression with corticosteroids is far from clear.^32^, ^36^ In the absence of any evidence to guide management, it seems reasonable to introduce cART (with a corticosteroid if the patient has a poor response) if vasculitic HIV-associated vasculopathy is suspected and other potential autoimmune or infectious causes have been excluded.

Evidence has convincingly shown that cART results in a reduction of all-cause mortality in patients with HIV.^108^, ^109^ Far less certain is whether cART treatment, particularly exposure to protease inhibitors, increases the long-term risk of stroke and myocardial infarction as a result of metabolic effects (eg, hypercholesterolaemia, already described) and extended survival (ageing is a risk factor for stroke and some populations infected with HIV have a high prevalence of cigarette smoking).^109^, ^110^, ^111^ The risk–benefit ratio of cART based on current knowledge seems to be favourable. However, in view of the concern about long-term stroke and cardiovascular disease risk, a pragmatic approach seems reasonable—ie, physicians should identify and manage risk factors, perhaps change the class of cART regimen, or consider a cholesterol-lowering drug if appropriate.^108^, ^109^, ^112^, ^113^, ^114^

None of the studies that guide the use of secondary prevention for stroke, including use of antiplatelets, statins, and blood-pressure-lowering therapy, can be directly extrapolated to patients with HIV who have had a stroke. However, general lifestyle factors and reduction of vascular risk factors seems sensible. Finally, the mode of HIV infection relevant to the patient should be considered, because this might affect underlying stroke risk factors, cause, and management. In sub-Saharan Africa, the major mode of HIV transmission is sexual intercourse among heterosexuals, whereas transmission via IDU is rare.^115^, ^116^ However, a history of IDU use is relevant, particuarly in areas where it is common, because it might be associated with several potential causes of stroke, including the use of specific drugs (cocaine, amphetamines, sympathomimetic drugs), infective endocarditis, and embolisation of particulate matter.^117^ In many regions, cigarette smoking is more common in people with HIV than in the general population.^118^

## Conclusions and future directions

Good community-based epidemiology to assess the burden and nature of HIV-related stroke is rare. Epidemiological studies with good clinical assessment of stroke patients, early imaging, clearly documented stroke types, subtypes, risk factors and causes, and importantly autopsy confirmation or exclusion of stroke and identification of the underlying cause of stroke, are needed in both high-income and low-income regions (panel 2). Improved knowledge about the mechanisms and causes of stroke should lead to improved investigation and treatment of patients. Further study is needed to assess the benefit and safety of acute stroke therapy and secondary stroke prevention in patients with HIV. Finally, the long-term effect of cART on stroke incidence is also unknown, and it might take years to establish this risk.

Panel 2 **Future directions and research priorities in HIV and stroke**

**Epidemiological, pathological, and clinical**

• Community-based epidemiology in low-income and high-income regions with detailed clinical assessment of stroke patients; early brain imaging; clearly documented stroke types and subtypes; assessment of risk factors and causes; autopsy confirmation or exclusion of stroke and identification of cause of stroke; assessment of the long-term effect of combined antiretroviral therapy (cART)
• Community-based case control studies to assess risk of stroke associated with HIV infection
• Assessment of risk and causes of recurrent stroke in patients with HIV-related stroke
• Outcome of thrombolysis treatment in patients with HIV infection who have had a stroke
• Post-mortem and biopsy evidence in particular to better define the nature of all forms of HIV-associated vasculopathy in stroke
• Assessment of the effect of HIV clades (or subtypes) on clinical presentation of stroke and HIV-related vascular disease
• Assessment of the effect of cofactors related to the mode of HIV transmission, such as associated intravenous drug misuse, on stroke cause and presentation

**Establishing pathogenesis and pathophysiology**

• Assessment of the effect of HIV infection and cART on the cerebral vascular endothelium (biological and mechanistic studies)
• Development of improved, ideally simple and cost-effective, diagnostic techniques to test for opportunistic infections associated with stroke in patients with HIV
• Assessment of the effect of infection burden (HIV and opportunistic infection) on accelerated atherosclerosis (deep sequencing methods to identify unknown pathogens)^119^

**Treatment**

• Development and assessment of optimum treatment strategies for management of stroke risk factors in patients on cART
• Development of antiretroviral therapy without the metabolic and endothelial effects of current drugs (provided it is as effective as current therapy)

Stroke is increasing in low-income and middle-income countries, and HIV infection—often prevalent in resource-poor regions—might add to this risk, although data are very scarce. Although cART might decrease stroke risk in the short term, its effect on the vasculature and long-term stroke risk is unknown, but could be substantial. Certainly, cART has increased the lifespan of those infected with HIV, but paradoxically it might increase their risk of stroke in the long term, as a result of endothelial and metabolic side-effects. The risk of stroke in patients with HIV and the need for further research to clarify burden, causes, pathogenesis, and management continues in areas with little or inadequate antiretroviral therapy. Equally, the need exists in high-income regions with patients on long-term antiretroviral therapy (ie, cART). The added stroke burden to patients and health services in all regions might only be realised in future decades.

## Search strategy and selection criteria

We identified references for this Review by searching Medline and PubMed for articles published in English between Jan, 1966 and May, 2012, using the terms, “cerebrovascular disorders”, “stroke”, “intracranial arteriosclerosis”, “arteriosclerosis”, “intracranial embolism”, “subarachnoid haemorrhage”, “intracranial haemorrhage”, “cerebral haemorrhage”, “endothelium”, “vascular disease”, “vasculitis”, “CNS vasculitis”, “vasculopathy”, or “atherosclerosis” in combination with “human immunodeficiency virus” [term exploded]. Addition of the term “AIDS” did not yield any further publications. Articles were also identified through the reference lists of selected publications and a search of the Cochrane Database. Only articles published in English were included. We used the WHO definition of stroke: rapidly developing signs of focal (or global) disturbance of cerebral function, leading to death or lasting longer than 24 h, with no apparent cause other than vascular.^120^ This definition includes ischaemic stroke, cerebral haemorrhage, and subarachnoid haemorrhage. We included manuscripts that describe pathological changes of stroke and potential stroke mechanisms and noted this in the Review.
